# Development of esophagogastroduodenoscopy in China: results from the national census in 2013 and 2020

**DOI:** 10.3389/fonc.2024.1366706

**Published:** 2024-06-07

**Authors:** Siwei Zhou, Zheran Chen, Yunfei Jiao, Zhiyuan Cheng, Ye Gao, Tianjiao Wang, Lei Xin, Rong Wan, Luowei Wang

**Affiliations:** ^1^ Department of Gastroenterology, Changhai Hospital, Naval Medical University, Shanghai, China; ^2^ National Digestive Endoscopy Improvement System, Shanghai, China; ^3^ Department of Gastroenterology, Shanghai General Hospital, Shanghai Jiao Tong University School of Medicine, Shanghai, China

**Keywords:** esophagogastroduodenoscopy, quality improvement, national census, China, upper gastrointestinal cancer

## Abstract

**Background:**

Given the significant burden of upper digestive diseases, there has been a substantial increase in the utilization of esophagogastroduodenoscopy (EGD) in China from 2012 to 2019. The objective of this study is to investigate the development, practice, and factors influencing the widespread use of EGD during this period.

**Methods:**

Two national censuses were conducted among all hospitals in mainland China that perform gastrointestinal endoscopy. These censuses aimed to extract information on the infrastructure, volume, and quality of EGD. The analysis of potential factors influencing EGD practice was based on real-world data from open access sources.

**Results:**

From 2012 to 2019, the number of hospitals performing EGD in mainland China increased from 1,518 to 2,265 (1.49-fold) in tertiary hospitals and from 3,633 to 4,097 (1.12-fold) in secondary hospitals, respectively. The national utilization rate of EGD also increased from 1,643.53 to 2,018.06 per 100,000 inhabitants, indicating a 1.23-fold increase. Regions with more endoscopists per 100,000 inhabitants (OR 9.61, *P*<0.001), more tertiary hospitals performing EGD per million inhabitants (OR 2.43, *P*<0.001), higher incidence of esophageal and gastric cancer (OR 2.09, *P*=0 016), and higher number of hospitals performing EGD per million inhabitants (OR 1.77, *P*=0.01) tended to provided more numerous and qualitied EGD. And hospital grading, regional GDP, incidence of esophageal and gastric cancer and the volume of EGD were observed as the significantly relevant factors of malignant dictation rate (MDR) (*P*<0.05), but not the number and educational background of endoscopists.

**Conclusion:**

Over the past seven years, China has made significant progress in EGD. However, challenges persist in terms of quality and inequality.

## 1 Introduction

Digestive diseases have emerged as a significant global public health concern due to their widespread impact. In 2019 alone, there were 1.8 million new cases and 1.46 million deaths attributed to esophageal and gastric cancer. Additionally, benign upper digestive system diseases accounted for 344 million new cases and 276.3 thousand deaths (1). A closer look at the situation in China reveals that this country faced 649 thousand new cases and 482 thousand deaths, accounting for 41.98% and 43.74% of the total new cancer cases and deaths, respectively (2). Among all types of cancers, digestive diseases rank third in terms of incidence and second in terms of mortality (3). Moreover, the aging population in China continues to steadily increase, and this demographic shift is considered a major contributing factor to the burden of esophageal and gastric cancer (4). Given this context, it is clear that the disease burden remains substantial in China and requires continued attention and resources.

In Western countries, digestive diseases are more prevalent in the lower digestive tract compared to the upper digestive tract (2). On the other hand, China has a higher prevalence of upper digestive tract diseases, such as esophageal and gastric cancers. The advancements in esophagogastroduodenoscopy (EGD) have played a crucial role in the diagnosis and treatment of these upper gastrointestinal diseases (5, 6). Performing EGD can effectively reduce the incidence and mortality of esophageal and gastric cancer in the population (7). In 2015, the national gastroenterology quality improvement system (NGQIS) of China was established, and nationwide surveys has been conducted to enhance the quality of upper gastrointestinal endoscopy (8). Additionally, specific indicators have been developed to improve the quality of these procedures in China (9). Furthermore, a long-term follow-up study in China revealed that for every additional RMB 8,448 invested in endoscopic screening, one gastric cancer death can be prevented. This highlights the effectiveness of endoscopic screening in early tumor prevention and treatment (10, 11). To significantly reduce the incidence and mortality rates associated with these cancers, policymakers must prioritize the promotion of the availability and quality of EGD.

The objective of this research is to offer a thorough analysis and evaluation of Chinese national progression in EGD, utilizing data from two countrywide surveys. The outcomes of this study will serve as crucial guidelines for policymakers to detect domains necessitating enhancements and proficiently establish standardized principles for EGD services in forthcoming years.

## 2 Method

### 2.1 Data resource

In order to investigate the performance of hospitals conducting endoscopy procedures in mainland China, the Chinese Digestive Endoscopy Census was carried out in 2013 and 2020. These comprehensive censuses covered all 31 provincial-level administrative regions. Following the retrieval of questionnaires, a random sample comprising over 10% of hospitals and reports was manually validated to ensure accuracy. In 2019, the number of tertiary and secondary hospitals performing EGD in mainland China increased to 2,265 and 4,097 (1518 and 3663 in 2012), respectively. The statistical data pertaining to EGD in this study were solely derived from the responses provided in these questionnaires (12). Moreover, diverse real-world data from multiple sources were gathered to determine the potential factors that influence the practice of EGD. The population at the national and provincial levels in 2012 and 2019 was obtained from the National Bureau of Statistics. The per capita gross domestic product (GDP) and population figures in regions with cancer registers were derived from publicly available statistical data and financial statements. The social-demographic index (SDI) of the 31 provincial regions was extracted from the Global Burden of Disease Results. Furthermore, data on the incidence of new cases and mortality rates related to esophageal and gastric cancer were acquired from the China Cancer Registry Annual Report in 2019.

### 2.2 Statements and definitions

In mainland China, hospitals are classified into Three levels based on their size and medical quality: tertiary, secondary, primary, and ungraded. The ownership of hospitals includes both public and private institutions. The education levels received by Chinese endoscopists mainly include bachelor’s and below, master’s and doctoral degrees. In this survey, a doctorate represents the highest educational background. This study defines the term ‘volume’ as the overall number of cases that underwent EGD for screening, surveillance, diagnosis, and treatment purposes. The malignancy detection rate (MDR) was determined by calculating the percentage of patients in whom one or more malignancies were identified and removed during EGD, irrespective of the initial reason for the procedure. The International Classification of Diseases (ICD) tenth edition codes for malignancies in MDR are as follows: C15-C15.9, D00.1, D13.0, C16-C16.9, D00.2, D13.1 and D37.1. The MDR serves as an indicator to assess the quality of EGD procedures (8).

### 2.3 Statistical analysis

We presented categorical data as numbers and percentages in order to assess the representativeness of our analysis. To compare the data of typical regions with the data of the whole nation, we employed Student’s t-test. In order to determine heteroscedasticity and suitability for the slope index, we conducted a non-constant variance score test. To analyze the correlations between potential factors and regional utilization rates, we utilized two-sided Spearman’s test, which is a nonparametric correlation statistical test. Based on the statistically significant results of the Spearman’s test, we established a multiple logistic regression model. We included the value of MDR in a mixed linear model with potentially relevant parameters to estimate the factors influencing EGD quality. For statistical analysis, we utilized SPSS version 26.0 for Windows (IBM Corp., Armonk, NY, USA). To determine statistical significance, we considered a two-sided *P* value < 0.05.

## 3 Results

### 3.1 General status of hospitals and device for EGD

From 2012 to 2019, the number of hospitals performing EGD in mainland China increased from 1,518 to 2,265 (1.49-fold) in tertiary hospitals and from 3,633 to 4,097 (1.12-fold) in secondary hospitals, respectively. The average number of hospitals per 100,000 inhabitants also increased from 0.38 to 0.45. In more detail, tertiary hospitals accounted for 29.30% and 35.60% of the hospitals, while secondary hospitals accounted for 70.70% and 64.60%%. Secondary hospitals remained the largest contributors, but tertiary hospitals showed an increase in their contributions. It is worth noting that the majority of these hospitals were public hospitals. Furthermore, in 2019, there were a total of 3,7845 gastroscopes and 1,338 transnasal gastroscopes. More detailed information about the hospitals and devices can be found in **Table 1**.

**Table 1:** The characteristics of tertiary and secondary hospitals providing esophagogastroduodenoscopy services in China in the years 2012 and 2019.

	2012			2019		
	Tertiary hospitals	Secondary hospitals	total*	Tertiary hospitals	Secondary hospitals	total*
All hospitals in mainland China	1,624	6,566	8,190	2,749	9,687	12,436
Hospitals performing EGD	1,518	3,663	5,181	2,265	4,097	6,362
Hospital characteristic						
Hospital ownership						
State-owned hospitals	1,406	2,816	4,222	2,148	3,579	5,727
Private hospitals	112	847	959	117	518	635
Hospital category						
General hospitals	1,447	3,601	5,048	2,020	3,654	5,674
Specialized hospitals	71	62	133	245	443	688
Devices						
Gastroscopes	–	–	16,491	26,349	11,496	37,845
Transnasal gastroscopes	–	–	–	1,104	234	1,338
Endoscopists	9,570	4,378	13,948	22,563	13,977	36,540
EGD volume(/10,000)	1,563.66	661.75	2,225.41	1,955.54	869.85	2,825.38
EGD under sedation(/10,000)	–	–	–	866.48	322.18	1,188.66
EGD quality						
MDR	–	–	–	2.17%	2.27%	2.20%
Early detection rate of esophageal cancer	15.53%	19.22%	16.61%	18.85%	17.41%	18.38%
Early detection rate of gastric cancer	17.49%	15.22%	16.86%	18.38%	15.42%	17.46%

*Total hospitals refer to the actual number of secondary and tertiary hospitals in China in 2019.

The total number of hospitals performing EGD increased by 23% from 2012 to 2019, but the percentage of these hospitals among all hospitals in mainland China decreased from 63.26% to 51.16%. Among secondary hospitals, the percentages were 55.79% and 42.29% and 93.47% and 82.39% among tertiary hospitals.

### 3.2 EGD volume and painless EGD

The total volume of EGD procedures in mainland China increased from 22.25 million cases to28.25 million cases, representing a 1.26-fold increase. Furthermore, the rate of EGD procedures per 100,000 inhabitants significantly rose from 1,643.53 to 2,018.06. In 2012 and 2019, tertiary hospitals accounted for 70.26% and 69.21% of the EGD volume, while secondary hospitals performed 29.74% and 30.79% respectively.

In 2019, 42.07% of the total EGD volume was performed with sedation. Among these cases, 72.9% were conducted in tertiary hospitals and 27.1% in secondary hospitals. Additionally, 41.1% and 57.4% of EGD procedures with sedation took place in public and private hospitals respectively. The sedation rate was 849.01 per 100,000 inhabitants.

### 3.3 Utilization rate and inequality of EGD service

**Figure 1:**
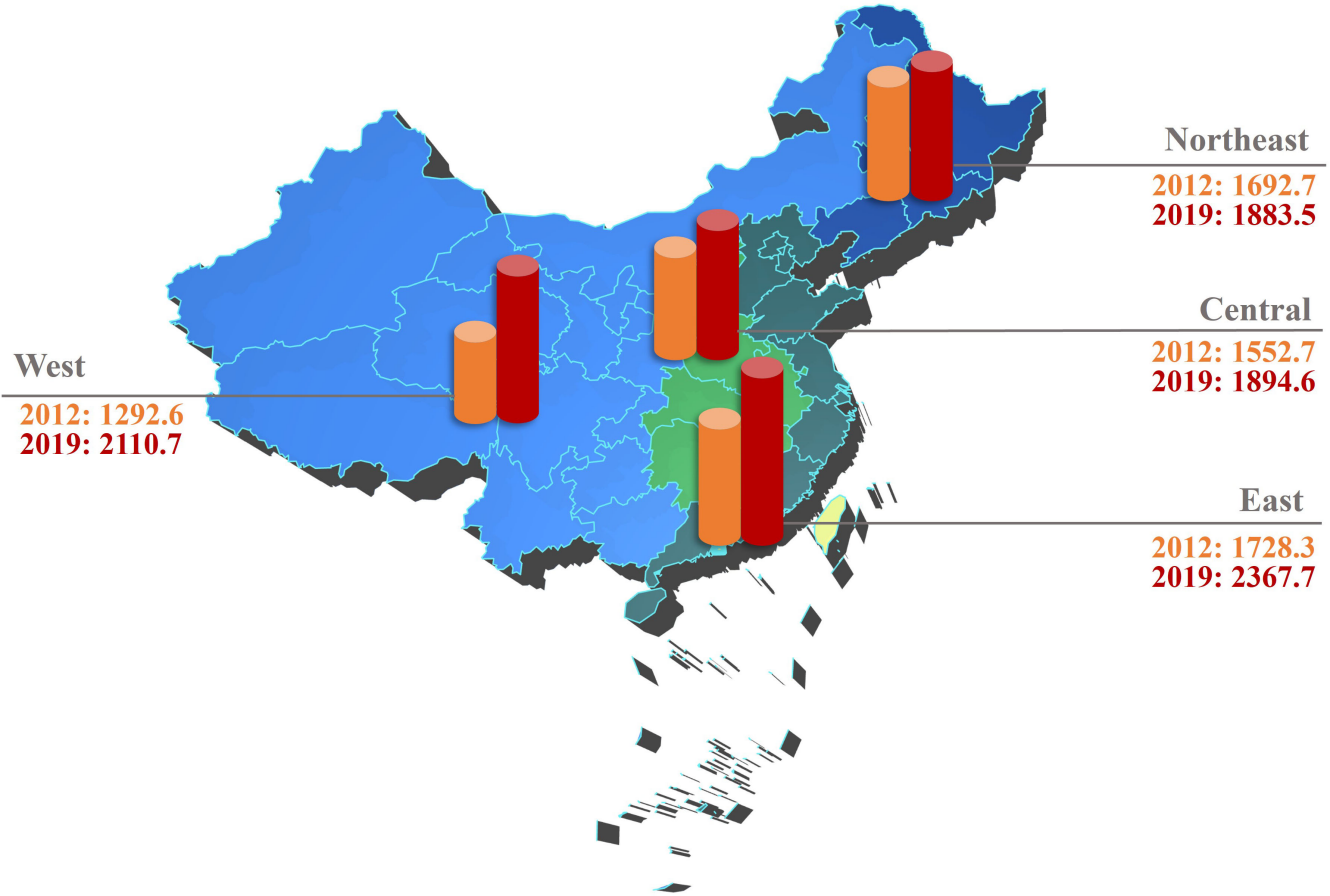
Development of EGD utilization (the number of EGD procedures per 100,000 inhabitants) in the 31 provincial regions of mainland China between 2012 and 2019 (East China includes Beijing, Shanghai, Tianjin, Hebei, Shandong, Jiangsu, Zhejiang, Fujian, Guangdong, and Hainan. Northeast China includes Heilongjiang, Jilin, and Liaoning. Central China includes Anhui, Henan, Hubei, Hunan, Jiangxi, and Shanxi. West China includes Chongqing, Gansu, Guangxi, Guizhou, Yunnan, Inner Mongolia, Ningxia, Qinghai, Shaanxi, Sichuan, Tibet, and Xinjiang). EGD, esophagogastroduodenoscopy.

The national utilization rate of EGD increased from 1,643.53 to 2,018.06 per 100,000 inhabitants (1.23-fold). Additionally, the regional utilization rates in every province of Chinese mainland also showed improvement (**Figure 1**). In 2012, the regional utilization rates of EGD were found to be correlated with regional SDI (ρ=0.384, *P*=0.033), but this correlation was not significant in 2019 (*P*=0.824).

To evaluate the potential factors influencing the regional utilization rates in the real world, a multiple logistic regression analysis was conducted using parameters derived from the statistically significant results mentioned in **Table 2**. The analysis revealed that higher utilization rates were observed in areas with a higher number of endoscopists per 100,000 inhabitants (OR 9.605, 95%CI 5.57–16.58; *P*<0.05) (**Table 2**).

**Table 2:** Multiple logistic regression analysis of factors associated with the regional utilization rate.

Variable	OR	95% CI	*P*
Incidence of esophageal and gastric cancer	2.087	1.148–3.795	0.016
Hospitals per million inhabitants	1.769	1.147–2.728	0.010
Tertiary hospitals per million inhabitants	2.432	1.714–3.450	<0.001
Endoscopists per 100,000 inhabitants	9.605	5.565–16.578	<0.001

The utilization rates were regarded as dichotomous variable based on the national average.

### 3.4 EGD quality and factors affecting MDR

Across the country, there was a significant improvement in the quality of EGD. The early detection rate of esophageal cancer rose from 16.61% in 2012 to 18.38% in 2019, and the early detection rate of gastric cancer increased from 16.86% to 17.46%.

In the multivariate mixed model, the hospital grading (ungraded, β=-0.007054), GDP per capita (β=-0.000357), incidence of esophageal and gastric cancer (β=19.310795)and the volume of EGD (β= -0.000014) showed a significant correlation with the MDR in different hospitals (*P*<0.05). However, the number of endoscopists (*P*=0.348) and the ratio of master’s and doctoral degrees (*P*=0.997) were found to be irrelevant to the EGD quality (**Table 3**).

**Table 3:** Mixed linear model of factors associated with MDR.

Parameters	β	95% CI	*P*
Intercept	0.018215	0.012614–0.023817	<0.001
Tertiary hospital	0.003385	-0.002061–0.008831	0.223
Secondary hospital	0.000802	-0.003914–0.005519	0.739
Ungraded hospital	-0.007054	-0.013661–0.000446	0.036
Incidence of esophageal and gastric cancer per 100,000 inhabitants	19.310795	14.619998–24.001593	<0.001
GDP per capita (per 10,000 RMB)	-0.000357	-0.000592–0.000122	0.003
Volume of EGD (per 100 cases)	-0.000014	-0.000071–0.000012	0.006
Number of endoscopists	0.000141	-0.000154–0.000436	0.348
Percentage of endoscopists with master’s and doctoral degree	0.000012	-0.005702–0.005726	0.997

The parameters of primary hospitals are redundant in the mixed linear model.

GDP, Gross Domestic Product; EGD, esophagogastroduodenoscopy.

## 4 Discussion

This study conducted an analysis and evaluation of the current status and characteristics of EGD services in China through two surveys on endoscopy practices. The findings indicated that hospital grading, GDP per capita, and incidence of esophageal and gastric cancer are significant factors influencing the MDR. The study offers valuable baseline data and intervention strategies for policymakers to mitigate regional disparities in EGD services.

Since 2005, the Chinese government has implemented the Cancer Screening Program in Rural and Urban areas to improve early diagnosis and treatment rates (4). Endoscopic screening has shown to reduce the occurrence and mortality of esophageal and gastric cancer (13). These efforts have particularly enhanced the EGD service in western China. Despite progress, China’s EGD utilization rate lags behind countries like Japan and the United States (14, 15). Our analysis found that regions with more tertiary hospitals, higher cancer incidence, more endoscopists, and more hospitals offering EGD services had higher utilization rates. However, disparities in EGD service provision persist, underscoring the need for policies targeting these factors and prioritizing underserved areas. Addressing the low sedation ratio during EGD procedures is also crucial, as China’s sedation rate of 42.1% is significantly lower than other countries, potentially impacting patient willingness to undergo the examination (16–18). China has made advancements in the utilization of EGD, however, the quality still falls short in comparison to developed nations.

Indicators such as NDR, early esophageal cancer detection rate, and early gastric cancer detection rate are commonly used to assess the effectiveness of EGD (19, 20). However, in this study, due to considerations regarding data collection feasibility and regression analysis requirements, we opted for MDR as an alternative measure. MDR, like NDR, focuses on monitoring the detection of malignant lesions during gastroscopy and yields a similar evaluation outcome.

A study examining colonoscopy quality discovered that the SDI was linked to the adenoma detection rate (ADR) during the procedure (21). This study investigated the correlation between SDI and MDR in 2012, which was initially identified. However, the correlation was no longer evident in the 2019 survey analysis, possibly attributed to decreased economic influences resulting from the growing prevalence of EGD examinations. Furthermore, a multiple logistic regression analysis revealed that GDP per capita did not influence EGD utilization. It is important to highlight that these results should not be viewed as definitive proof. Further research and data collection are necessary to fully understand the relationship between economic development levels and EGD utilization.

Strengthening basic and primary healthcare services is crucial to enhance and expand EGD services. Tertiary hospitals play a significant role in both the quantity and quality of EGD procedures. While secondary and primary hospitals may perform fewer EGD surgeries with lower quality, they offer greater accessibility and quantity. As the population ages, the incidence of esophageal and gastric cancer is expected to rise, increasing the demand for EGD procedures (4). Therefore, enhancing the performance of EGD procedures in basic hospitals is essential to alleviate this growing burden.

There are inherent limitations in this study. Primarily, the retrospective nature of the census design introduces the possibility of recall bias, which cannot be completely eliminated. Secondly, the data collected only includes the overall volume of EGD, lacking clear classification for screening, surveillance, and treatment objectives. Thirdly, The definition of MDR solely encompasses esophageal cancer and gastric cancer, excluding precancerous lesions, gastroesophageal junction tumors, and duodenal tumors. This limitation leads to a lower estimated value of MDR compared to NDR, attributed to challenges in accurately capturing this information during investigations. Lastly, the utilization rates of other countries were estimated based on studies and reports with diverse designs and coverage, thereby restricting the scope of accurate comparison to a general overview.

To conclude, comprehending the national approach to upper gastrointestinal endoscopy is a crucial initial phase in establishing enhancement of quality. The outcomes we obtained will be a valuable point of reference for formulating an efficient nation-specific examination plan for upper gastrointestinal illnesses. At present, China encounters various prospects and obstacles regarding gastrointestinal endoscopy, and immediate measures at the policy and hospital levels are imperative.

## Data availability statement

The raw data supporting the conclusions of this article will be made available by the authors, without undue reservation.

## Author contributions

SZ: Formal analysis, Writing – review & editing. ZRC: Investigation, Software, Writing – original draft. YJ: Writing – original draft, Data curation, Formal analysis. ZYC: Data curation, Investigation, Writing – original draft. YG: Data curation, Writing – original draft. TW: Investigation, Writing – review & editing. LX: Data curation, Writing – review & editing. RW: Conceptualization, Resources, Writing – review & editing. LW: Conceptualization, Funding acquisition, Writing – review & editing.
